# Microfilaremic *Dirofilaria repens* Infection in Patient from Serbia

**DOI:** 10.3201/eid2912.230796

**Published:** 2023-12

**Authors:** Suzana Tasić-Otasevic, Milan Golubović, Simone Trichei, Dragan Zdravkovic, Radojičić Jordan, Simona Gabrielli

**Affiliations:** University of Niš, Nis, Serbia (S. Tasić-Otasevic, M. Golubović);; Public Health Institute, Nis (S. Tasić-Otasevic, D. Zdravkovic);; University Clinical Center of Niš, Nis (M. Golubović);; Sapienza University of Rome, Rome, Italy (S. Trichei, S. Gabrielli);; Niš Military Hospital, Nis (R. Jordan)

**Keywords:** *Dirofilaria repens*, microfilariae, dirofilariasis, nematodes, humans, parasites, vector-borne infections, zoonoses, Serbia

## Abstract

We report a case of *Dirofilaria repens* infection causing microfilaremia in a patient from Serbia. Serum samples tested positive for *D*. *repens* IgG by ELISA. Our findings and those of others suggest the parasite's progressive adaptation to humans. Clinicians should be aware that microfilaremia can develop during *Dirofilaria* spp. infections.

*Dirofilaria repens* is a vectorborne filarial helminth of carnivores, mainly domesticated dogs (*1*). Humans are considered accidental hosts, in which the parasite induces local inflammation causing granulomatous reactions primarily detected in subcutaneous and ocular tissues. Because humans are not natural hosts, microfilariae are typically absent from peripheral blood; thus, diagnostic procedures require morphologic and molecular analyses of removed worms (*2*). Immunodiagnostic tests are being designed as potential alternatives to invasive diagnostic procedures (*3*). This parasite rarely evades the human host’s immune system to reach sexual maturity. The literature reports 22 cases of human *D. repens* microfilaremia, of which several have been confirmed through molecular examination (*3*,*4*). We describe a case of human dirofilariasis with circulating microfilariae in a patient from Serbia.

A 43-year-old professional soldier in the army of Serbia was first seen for a walnut-sized swelling accompanied by itching on the inner side of his thigh, which we promptly treated with ciprofloxacin (1 g/d) for 14 days. Two months after the initial swelling, the patient noted another similar protuberance on his inner thigh that migrated toward the back of the thigh every 2–3 days. An ultrasound detected a 13.5 × 8 mm subcutaneous nodule. Biochemical analyses of the patient’s blood and blood cell counts were within reference ranges, including eosinophil levels; however, surgical intervention was required 1 month after the ultrasound to excise the nodule and investigate its origin. Examination of the removed nodule revealed a *Dirofilaria repens*–like specimen. Twenty days after nodule removal, the percentage of eosinophils in the patient’s peripheral blood increased to 14%. We performed a modified Knotts test on EDTA blood, which revealed the presence of 2 microfilariae/mL. The mean microfilaria body length was 377–378 µm and mean width was 7.35–7.6 µm; they had no sheath but had obtuse cervical ends, 2–3 separate nuclei in the head space, and nuclei-free filiform tails (Figure).

**Figure:**
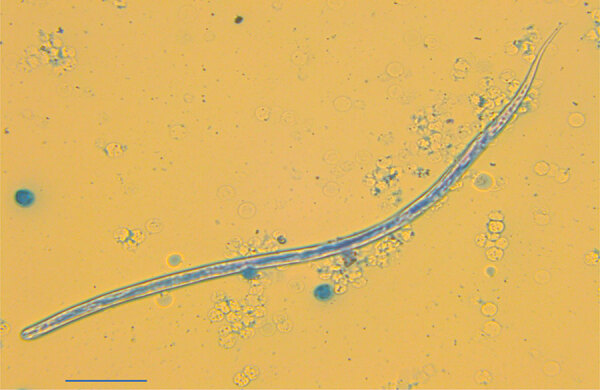
Microscopic image of *Dirofilaria repens* microfilaria in case study of microfilaremic *D*. *repens* infection in patient from Serbia. A blood sample from the patient was processed and stained with methylene blue. Scale bar indicates 200 μm.

We used species-specific PCR that amplified a portion of the cytochrome oxidase subunit 1 gene, *cox1*, to confirm the microfilariae were *D. repens* (Appendix). BLAST (https://blast.ncbi.nlm.nih.gov) analysis of the nucleotide sequence revealed a 97%–100% identity with published sequences of *D. repens* (Appendix Figure). We deposited the sequence in GenBank (accession no. OR426928.1). We constructed a maximum-likelihood phylogenetic tree of *cox1* sequences from this study and representative *D*. *repens* isolates from animals and humans in Europe by using MEGA version 11 software (*5*) and the Kimura 2-parameter distance model. We assessed the robustness of nodes by using 500 bootstrap replicates; *Ascaris lumbricoides* (GenBank accession no. AB591801.1) was the outgroup (Appendix Figure).

We used ELISAs to detect *Dirofilaria* spp. IgG. Cutoff optical densities were 1.8 for the commercial *Acanthocheilonema viteae* IgG ELISA kit (Bordier, http://www.bordier.ch), which detects IgG against various filarial nematodes in human serum, and 2.3 for an in-house ELISA (Appendix).

Two months after the *D. repens* diagnosis, the patient had an eosinophil count within reference ranges. No further microfilariae were detected in peripheral blood smears during monitoring.

*Dirofilaria* spp. infections are increasing globally, posing a substantial threat to pets, particularly dogs (*1*). The rising number of human dirofilariasis cases underscores the need for large-scale epidemiologic studies to establish effective preventive measures (*1*,*4*). Although humans are generally regarded as unsuitable hosts for *D*. *repens*, detection of single subadult or adult worms in humans is not uncommon (*3*). The worms can infrequently develop into mature adults, mate, and produce microfilariae, which can potentially enter the bloodstream in human hosts. Diagnosis involves morphologic and molecular analyses, but surgical removal of the worm and specialized equipment are required (*2*). Serologic tests detect specific antibodies, providing a more reliable epidemiologic picture of the zoonosis within a study area (*3*). 

Several protocols are available to investigate seroreactivity in humans (*6*,*7*), but antibody responses might not be detected until the nematode has migrated internally from subcutaneous tissue (*8*). Localization of larvae in ocular tissue might confer greater protection for the parasite, leading to a low predictive positive value for diagnosing *Dirofilaria* spp. infection (*9*).

No established treatment protocols exist for dirofilariasis or microfilaremia, leaving therapeutic decisions to physicians. Surgery is the primary treatment, accompanied by adjuvant therapy options, such as ivermectin, albendazole, or diethylcarbamazine. Doxycycline, a treatment for *Wolbachia* endosymbionts, has been used (*9*). In this case, we considered anthelmintics after surgical removal and microfilariae detection but chose continuous monitoring. Two months after diagnosis, the patient’s health was satisfactory, and he had no symptoms or complications from the infection. 

In conclusion, we report a case of *D. repens* microfilaremia in a professional soldier frequently exposed to vector biting because of prolonged outdoor activities during military training in northern Serbia, a dirofilariasis-endemic area (*10*). The presence of circulating microfilariae highlighted by this and previous studies shows the parasite’s progressive adaptation to humans and suggests a potential role for humans as a definitive *D. repens* host. Although a role for humans as an infection reservoir remains to be clarified, clinicians should be aware that microfilaremia can develop during *Dirofilaria* spp. infections.

Appendix Additional information for microfilaremic *Dirofilaria repens* infection in patient from Serbia.
