# Supplementary material for: Microfilaremic Dirofilaria repens Infection in Patient from Serbia
Source: Emerg Infect Dis. 2023 Dec;29(12):2548–50. doi: 10.3201/eid2912.230796 (PMC10683817; doi:10.3201/eid2912.230796)
Supplement: Appendix — Additional information for microfilaremic Dirofilaria repens infection in patient from Serbia. [file 23-0796-Techapp-s1.pdf]

Article DOI: https://doi.org/10.3201/eid2912.230796

EID cannot ensure accessibility for supplementary materials supplied by authors. Readers who have difficulty accessing supplementary content should contact the authors for assistance.

Microfilaremic *Dirofilaria repens* Infection in Patient from Serbia

Appendix

Additional Methods

Knott concentration technique

The modified Knotts technique was used to concentrate and detect microfilariae as previously described (*1*). In brief, 1 mL of EDTA blood was mixed with 9 mL of distilled water in a 15 mL tube. The tube was gently inverted 4 times to mix the solution and then centrifuged for 3 min at 1500×g. The supernatant was poured off and 1–2 drops of 1% methylene blue were added. A drop of the sediment was placed on a glass slide and covered with a coverslip. The slide was examined under a microscope at magnification ×100 to assess the presence of microfilariae and at ×400 to observe morphologic features.

Molecular Analysis

An ≈650-bp fragment of the mitochondrial *cox1* gene locus was amplified by PCR using the primers COIintF (5′-TGATTGGTGGTTTTGGTAA-3′) and COIintR (5′-ATAAGTACGAGTATCAATATC-3′) (*2*,*3*). PCR was performed in a final volume of 25 µL containing 2 µL of extracted DNA, 2.5 µL of 10X buffer, final concentration of 1.5 mM $MgCl_2$, 0.2 mM of each deoxynucleotide triphosphate, 1 mM of each primer, and 1 unit of BIOTAQ DNA polymerase (Bioline, https://www.bioline.com), diluted to 25 µL with double-distilled water. To test the specificity of the reaction, 2 µL of DNA extracted from a known *Dirofilaria repens* specimen was used as a positive control, and 2 µL of double-distilled water was included as a negative control in each PCR run. The amplification was performed in a thermocycler (Bio-Rad Laboratories, https://www.bio-rad.com) by using the following cyclic program: initial denaturation at 94°C for 10 min; then 5 cycles of further denaturation at 94°C for 30 s, annealing

at 52°C for 45 s, and extension at 72°C for 1 min; then 30 cycles of further denaturation at 94°C for 30 s, annealing at 54°C for 45 s, and extension at 72°C for 1 min; then a final extension for 7 min at 72°C. The PCR products were separated on a 1.2% agarose gel stained with SafeView (NBS Biologicals, https://www.nbsbio.co.uk) that underwent electrophoresis at 100 volts for 45 min and was visualized on a UV transilluminator (Bio-Rad Laboratories). An amplicon of the expected size was directly sequenced by Bio-Fab Research (https://www.biofabresearch.com). The resulting chromatogram was analyzed and edited by using Chromas version 2.33 software (Technelysium Pty Ltd, https://technelysium.com.au). The nucleotide sequence was compared with previously published *D. repens* sequences deposited in GenBank by using BLAST (https://blast.ncbi.nlm.nih.gov).

Phylogenetic Analysis

The maximum-likelihood phylogram comparing the *cox1* sequence from this study and representative *D. repens* isolates from animals and humans in Europe was constructed by using MEGA version 11 software and the Kimura 2-parameter distance model. The robustness of nodes was assessed by using 500 bootstrap replicates; *Ascaris lumbricoides* (GenBank accession no. AB591801.1) was the outgroup (Appendix Figure).

In-House ELISA

Serum samples collected from the patient were analyzed by using an ELISA that was prepared in-house and incorporated somatic antigens of *D. repens*. In brief, worms obtained from necropsy of naturally infected dogs were washed, macerated, and sonicated (3 cycles at 70 kHz, 30 s per cycle) in sterile saline solution. The homogenate was centrifuged at 16,000×g for 30 min. The supernatant was dialyzed against 0.01 M phosphate-buffered saline, pH 7.2. The protein concentration was measured by using the Bradford method, and an ELISA microplate was coated with antigens at a final concentration of 0.8 µg/µL. Using the protocol performed in a previous survey (*4*), serum was tested in the solid-phase ELISA at a dilution of 1:80 to detect *D. repens* IgG. Goat anti-human IgG conjugated to horseradish peroxidase (Sigma-Aldrich, https://www.sigmaaldrich.com) was used as a secondary antibody at a 1:40,000 dilution. Optical density was measured at 492 nm on an Easy-Reader instrument (Bio-Rad Laboratories). The cutoff point (optical density = 1.03) was established by calculating the mean value $\pm$3 SD of 30 serum samples obtained from clinically healthy humans (negative controls).

References

1. Genchi M, Ciuca L, Vismarra A, Ciccone E, Cringoli G, Kramer L, et al. Evaluation of alternative reagents on the performance of the modified Knott's test. Vet Parasitol. 2021;298:109555. PubMed https://doi.org/10.1016/j.vetpar.2021.109555

2. Casiraghi M, Anderson TJ, Bandi C, Bazzocchi C, Genchi C. A phylogenetic analysis of filarial nematodes: comparison with the phylogeny of *Wolbachia* endosymbionts. Parasitology. 2001;122:93–103. PubMed https://doi.org/10.1017/s0031182000007149

3. Casiraghi M, Bain O, Guerrero R, Martin C, Pocacqua V, Gardner SL, et al. Mapping the presence of *Wolbachia pipientis* on the phylogeny of filarial nematodes: evidence for symbiont loss during evolution. Int J Parasitol. 2004;34:191–203. PubMed https://doi.org/10.1016/j.ijpara.2003.10.004

4. Tasić-Otašević SA, Gabrielli SV, Tasić AV, Miladinovićtasić NL, Kostić JT, Ignjatović AM, et al. Seroreactivity to *Dirofilaria* antigens in people from different areas of Serbia. BMC Infect Dis. 2014;14:68. PubMed https://doi.org/10.1186/1471-2334-14-68

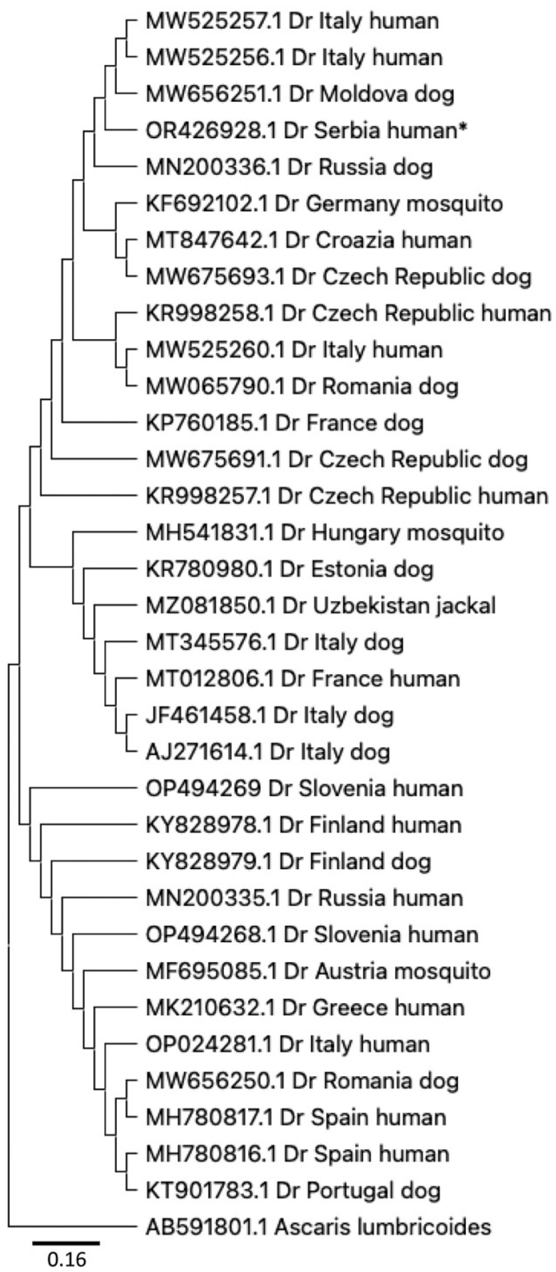

Appendix Figure. Phylogenetic analysis of partial *cox1* gene sequences from *Dirofilaria repens* detected in this study and from representative *D*. *repens* isolates from animals and humans from Europe. Evolutionary analysis was conducted by using the maximum-likelihood method and Kimura 2-parameter model with discrete Gamma distribution to model evolutionary rate differences among sites selected by a best-fit model. The robustness of nodes was assessed with 500 bootstrap replicates; *Ascaris lumbricoides* (GenBank accession no. AB591801.1) was the outgroup. Asterisk indicates the *D*. *repens* sequence from this study (GenBank accession no. OR426928.1). GenBank accession numbers and host species for all sequences are indicated. Scale bar indicates nucleotide substitutions per site.
